# Can professionals “keep the tiller straight” in organizations? Resistance to reframing and decoy alternatives in workplace decision-making

**DOI:** 10.3389/fpsyg.2024.1270012

**Published:** 2024-02-28

**Authors:** Laura Angioletti, Carlotta Acconito, Davide Crivelli, Michela Balconi

**Affiliations:** ^1^International research center for Cognitive Applied Neuroscience (IrcCAN), Catholic University of the Sacred Heart, Milan, Italy; ^2^Research Unit in Affective and Social Neuroscience, Department of Psychology, Catholic University of the Sacred Heart, Milan, Italy

**Keywords:** reframe resistance, decoy effect, behavioral decision-making, organization, professionals

## Abstract

So far, little is known about the ability to contrast contextual bias as a protective factor in an ever-changing organizational environment. This study assessed whether professionals with different seniority can resist the reframing and the decoy effect under decision-making conditions and whether decision-making styles can predict the resistance to such covert influence tactics. To reach this aim, two groups of professionals divided into senior and junior professionals performed two novel tasks, a Resistance to Reframe Task (RRT) and a Resistance to Alternatives Task (RAT), which, by including ecological scenarios that represent typical decision situations that could arise in the company, can measure the resistance to such covert influence tactics. Decision-making styles were measured through the General Decision-Making Style (GDMS) and the Maximization Scale (MS). Results showed that all professionals were able to resist more to the reframing (at the RRT) than the decoy alternatives (RAT), without any difference between groups. In addition, higher GDMS-dependent subscale scores predict lower RRT scores, especially in the group of senior professionals. However, in the group of junior professionals, the GDMS-dependent subscale and MS high standards subscale predicted lower RAT scores. To conclude, this study showed that professionals know how to “keep the tiller straight” in organizations, especially when facing reframing conditions, rather than decoy alternatives; however, the predominance of dependent decision-making styles (for both senior and junior professionals) and the tendency to hold high standards in decisions (mainly for juniors) could undermine their resistance capacity and make them vulnerable to these covert influence tactics.

## 1 Introduction

Current working conditions are characterized by a post-pandemic working modality involving many changes in the organization of work (Chan et al., 2023). As a result, employees—both senior professionals and novices—may experience a great deal of uncertainty as they adapt to a new way of working. Organizations are becoming more aware that they must invest in the wellbeing of employees, and, within this framework, we have recently argued the importance of professionals’ neurocognitive health, giving particular attention to the assessment, and strengthening of executive functions (EFs) in the workplace (Balconi et al., 2020).

In this study, we seek to test whether, compared to professionals already entered the world of work, junior professionals can resist contextual bias, such as the reframing and the decoy effect under decision-making conditions. Moreover, we aim to investigate whether there is a decision-making style that predicts such resistance to reframing and yielding in the decoy effect.

We did so by asking a sample of junior and senior professionals to perform two novel tasks, a Resistance to Reframe Task (RRT) and a Resistance to Alternatives Task (RAT), which, by including ecological scenarios that represent typical decision situations that could arise in the company, in which the professionals were asked to identify themselves, can measure their resistance to such covert influence tactics. During the tasks, behavioral responses were collected to compose specific behavioral indices of RRT and RAT.

Furthermore, the General Decision-Making Scale (GDMS; Scott and Bruce, 1995) and the Maximization Scale (MS; Nenkov et al., 2008) were applied to profile professionals’ decision-making styles and explore potential associations between the five different GDMS decision-making styles (rational, intuitive, avoidant, dependent, and spontaneous), the MS subscales (high standard, alternative search, and decision difficulty), and the ability to resist reframing and alternatives, as an high-order executive control ability.

Indeed, EFs consist of a family of high-order cognitive functions (including working memory, cognitive flexibility, inhibitory control, decision-making, and other functions) that are the basis for the management of sustained attention, the control of impulsive reactions control, support goal-attainment, and are especially relevant for promoting a quick and flexible adaptation to shifting environmental demands (Miller and Cohen, 2001; Diamond, 2013). Among EFs, decision-making plays a crucial role at all professionals’ levels (Del Missier et al., 2010; Balconi, 2023; Rovelli and Allegretta, 2023), especially under conditions of uncertainty, which can affect both experienced and junior professionals. If on the one hand, the ability to be flexible in decisions and adapt to changes has been valued (Laureiro-Martínez and Brusoni, 2018); on the other hand, it proves useful for professionals to be able also to maintain one’s decision independently from the context (to use an idiom, “to keep the tiller straight” while navigating organizations), for instance, despite a situation being subjected to covert influence tactics that act on the context, such as reframing strategy or decoy effect.

The strategy of reframing, in the therapeutic context, refers to a type of interpretation that provides a new meaning or frame of reference to perspectives in a constructive direction (i.e., positive reframing) (Guterman, 1992; Bateson, 1995), by often drawing positive implications from adverse circumstances. On the contrary, negative reframing provides helpful warnings about difficult situations (Tracy et al., 2002). This concept has been exploited in communication and political studies (Catellani and Bertolotti, 2017; Voelkel et al., 2023), reaching up to be used in the organizational field for enabling professionals to see organizational issues through different lenses (Winter et al., 1997; Bolman and Deal, 2017; Yilmaz et al., 2021).

Another context-dependent phenomenon is the decoy effect, which happens when additional alternatives proposed to the individual can change one’s previous choice (Huber et al., 1982). Regarding the link between EFs and resistance to reframing and the decoy effect, neuroscience studies demonstrated how additional cognitive control is needed to inhibit the automatic process derived from the decoy effect (Hu and Yu, 2014) and contrast the framing effect (De Martino et al., 2006; Xu et al., 2013).

To the best of our knowledge, the influence of reframing strategy on professionals’ decision-making has never been tested before, as well as the ability of professionals to resist such covert influence tactics in the workplace.

On the contrary, the bias derived from adding a decoy alternative under decision-making conditions (i.e., the decoy effect) has been studied in organizations in relation to hiring decisions (Slaughter et al., 2011; Keck and Tang, 2020). Interestingly, concerning differences between junior and senior professionals, Slaughter et al. (2011) examined the extent to which highly experienced executive Master of Business Administration Students (executives with more than 10 years of experience) and inexperienced undergraduate students (junior-level college students) can use the decoy effect as a covert strategy for influencing the outcome of selection decisions. The decoy effect happens in a situation when the inclusion of an inferior alternative (in this case a candidate) in a set of options alters the preference relationships between the current, superior options (i.e., change the attraction toward other candidates) (Huber and Puto, 1983). Authors showed that participants could similarly build an asymmetrical dominance set of candidates that generated a decoy effect and that students outperformed executives. The authors supposed that it is the type of expertise, rather than the amount of experience and age, that provides individuals with this skill.

Although the study by Slaughter et al. (2011) has the merit of deepening decision-making skills in the professional context, it has the limitation of using only the recruitment and selection scenarios (typical of human resources professional figures) perhaps unfamiliar to the participants. In addition, the authors did not find any significant relation between demographical information, job dimensions with selection decisions, or behavioral performance (Slaughter et al., 2011). Yet, they neglected the link between behavioral performance and individuals’ decision-making styles.

Thus, there appears to be an important gap in the literature, and filling this gap can provide an important missing link from the decision-making perspective (in terms of decision-making styles and resistance to these covert influences) to the organizational literature. Decision-making styles can be conceived as learned habit-based propensity to react in a specific way in a certain decision context (Scott and Bruce, 1995). Considered as individual differences in decision-making profiles, they were classically explored with validated questionnaires such as the General Decision-Making Scale (GDMS; Scott and Bruce, 1995), which proposes five different independent decision styles (rational, intuitive, avoidant, dependent, and spontaneous), or the Maximization Scale (MS; Nenkov et al., 2008), which include three main dimensions (the tendency to hold high standards, to seek better alternatives and the difficulty in deciding), and they have previously examined also in relation to different professions (Iannello, 2007).

Given these premises and considering the level of expertise and seniority, we hypothesize higher RRT and RAT indices in senior than junior professionals under decision-making conditions. In addition, it is supposed that MS high standards and alternative search subscale scores could be predictors of lower RAT scores in junior professionals, as the tendency to hold high standards for oneself and things in general, and the tendency to always seek better options can generate a greater tendency to yield (and thus resist less) to new alternatives, especially for junior professionals that are entering the world of work. Moreover, it is expected to find a relation between GDMS subscale scores and RRT and RAT scores. In particular, a GDMS-dependent decision-making style could predict lower RRT and RAT scores for both professional categories, as, regardless of seniority, this decision-making style is characterized by constantly seeking suggestions and advice from other people before deciding (Thunholm, 2004) and this may generate a lower ability to resist external influences deriving from a reframed situation or the presentation of multiple alternatives.

## 2 Methods

### 2.1 Participants

A total of 61 professionals (40 females and 21 males; age range = 22–60; Mage = 34.58 years; SD age = 11.44) participated in the study. Based on their age and expertise, the sample was divided into two groups: The first group consisted of a total of 32 junior professionals (Mage = 34.21 years; SD age = 11.32) at the beginning of their working experience, with a minimum expertise of 2 years and a maximum of 3 years in the same role (apprenticeship); the second group was composed of 29 senior professionals (Mage = 38.98 years; SD age = 10.87) already placed on the labor market, who hold a managerial role for at least 5 years. All participants were recruited from different organizations in Northern Italy between October 2022 and April 2023. They were all employed in managerial divisions and the same job position for approximately 2 years at the time of the experiment. This criterion was chosen to avoid potential biases derived from situational factors, such as the potential increase of stress due to a new job position or a greater workload while adjusting to new tasks or obligations (Balconi et al., 2023a, 2023b). Moreover, to increase the generalizability of the findings, professionals were recruited from various internal divisions (for example, human resource management, training and professional learning, engineering and maintenance management, service quality monitoring, infrastructure management, and others) to increase the variety of the sample in terms of professional specialization. In each of the two groups, the internal divisions were equally distributed.

Exclusion criteria were levels of depression, previous psychiatric or neurology disorders, and undergoing treatment with concomitant psychoactive drug therapy that could alter cognitive or decision-making abilities (Angioletti et al., 2023), as well as abnormal short- and long-term memory or low global cognitive functioning. The study was approved by the Ethics Committee of the Department of Psychology of the Catholic University of the Sacred Heart, Milan, Italy. The study was carried out under the Declaration of Helsinki Principles (2013). Written informed consent was obtained from the participants, and they were informed of their right to discontinue participation at any time.

### 2.2 Experimental procedure

**Figure 1:**
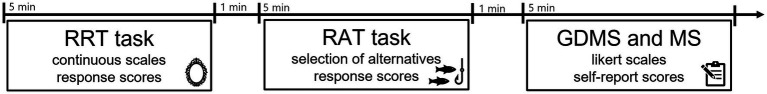
Experimental flow.

Participants sat in a quiet room located on their company site, in front of a computer place approximately 80 cm distant from them. After signing the written informed consent, they received the instruction for performing the two different tasks, RRT and RAT, administered via a web-based survey and experiment-management platform (Qualtrics XM platform; Qualtrics LLC, Provo, UT, USA). The GDMS and the MS were administered at the conclusion of the tasks to collect participants’ self-report data. The experiment lasted approximately 15 min (Figure 1).

#### 2.2.1 Resistance to reframe task (RRT)

In the Resistance to Reframe Task (RRT), the participants were presented with two different scenarios divided into two decisional steps. In both decisional steps, they were asked to identify themselves with the scene and choose the alternative that they thought was most suitable in a set of multiple options.

In the first decisional step, participants were presented with a script regarding a critical work situation in which they were asked to make a decision. For instance, in the first scenario, they were presented with the following script:

*“You must participate together with all the executives of your company in a particularly hard decision. Due to a funding cut, you must decide whether to close some plants and lay off some employees. You have 4 factories and 6,000 employees in total. Let us introduce you to some of the people who work in these establishments.”*

After the presentation of the script, it was shown to them the picture of the four companies and the four employees mentioned in the script.

They were asked to choose which of the four plants would they choose to keep open (selecting one of the four options presented) and to rate their confidence in the choice on a Likert scale from 1 to 5, where 1 corresponded to “not at all” and 5 to “entirely sure” (i.e., confidence rating in the first decisional step).

In the second decisional step, participants were then presented with the reframed part of the task, in which they were told that *“based on the choice they have made, the other employees will lose their jobs*” and they were then asked once again to rate their confidence in the choice on a Likert scale from 1 to 5, where 1 corresponded to “not at all” and 5 to “entirely sure” (i.e., confidence rating in the second decisional step, that was reframed).

After this first scenario, a second different scenario was also presented to the participants: The order of presentation was randomized and counterbalanced between participants.

Response scores were calculated based on the difference between the confidence rating in the first decisional step and the confidence rating in the second decisional step (the reframed one), averaged across scenarios. Such average difference scoring was, then, transcribed to a five-point scale based on the following rules:

- AvgDiff <1 ➔ RS = 5.
- 1 ≤ AvgDiff <2 ➔ RS = 4.
- AvgDiff = 2 ➔ RS = 3.
- 2 < AvgDiff <3 ➔ RS = 2.
- AvgDiff ≥3 ➔ RS = 1.

where AvgDiff stands for average difference scores as above described, and RS stands for response scores. A higher score corresponds to a higher ability to resist the reframe, while a lower score to a lower ability to resist it.

Response scores were, then, transcribed offline in deciles to compute the Resistance to the Reframe Task index (RRTi).

#### 2.2.2 Resistance to the alternatives task (RAT)

In the Resistance to the Alternatives Task (RAT), participants were presented with three different realistic decision-making scenarios related to purchases of basic facilities for the company (printer, chairs, and hard disks) and containing decoy alternatives. Participants were asked to identify themselves in those decisional scenarios and then to provide a behavioral response by choosing which of several proposed options they thought was most suitable for them.

Each scenario presented two decisional steps: In the first decisional step, participants could choose between two alternative options; in the second decisional step, a third superior alternative and the superior option were added. Table 1 reports the example of one scenario and related alternatives for each decisional step.

**Table 1:** Example of the printer scenario and its two decisional steps with the relative alternatives of choice.

RAT		
	First decisional step	Second decisional step
Printer scenario	Your company needs to buy six new office printers. You contact your supplier who offers you two alternatives: Express printers: single function printer, performs monochromatic printing operations in A4 format. Compact, fast, and reliable. Maximum savings on toner. Price €490 each Business printers: multifunction printer, performs color printing, copying, and scanning operations. An HD prints in A4 format. Intelligent and safe technology, with high-quality resolution. Price €820 each	Your company needs to buy 6 new office printers. You contact your supplier who offers you 2 alternatives: Express printers: single function printer, performs monochromatic printing operations in A4 format. Compact, fast, and reliable. Maximum savings on toner. Price €490 each Business printers: multifunction printer, performs color printing, copying, and scanning operations. HD prints in A4 format. Intelligent and safe technology, with high-quality resolution. Price €820 each Advanced Printers: multifunction printer, performs color printing, copying, scanning, and faxing. Professional prints on different formats and dimensions in very high definition. New-generation technology and innovative design. Price €1,250 each
Alternatives of choice	What do you choose? Express printers (€ 490) Business Printers (€ 820)	What do you choose? Express printers (€ 490) Business Printers (€ 820) Advance Printers (€ 1,250)

The ecological validity of the decision scenarios with their alternatives was taken into consideration during their creation and was pursued with realistic situations and problems referred to the organizational environment, with which professionals could easily identify. Each scenario created was validated by a panel of independent judges, who assessed its ecological validity as well as its realism and clarity. In addition, to avoid an order effect, each scenario and decisional step was presented in random order and counterbalanced between participants.

To calculate the response scores, a score of 1 was assigned if the choice matched the two decisional steps (i.e., the selection of the same alternatives in the two decisional steps of each scenario), while a score of 0 was assigned if a different choice was made (i.e., the selection of different alternatives in the two decisional steps of each scenario).

The scores assigned to each scenario were then summed to obtain a final score of resistance to the alternatives. A higher score corresponds to a higher ability to resist the alternatives, while a lower score to a lower ability to resist them. Response scores were, then, transcribed offline into deciles to compute the Resistance to the Alternative Task index (RATi).

### 2.3 Self-report scales for measuring decision-making style

The Italian version of the General Decision-Making Style (GDMS) (Scott and Bruce, 1995; Gambetti et al., 2008) and the Maximization Scale (MS) (Schwartz et al., 2002; Nenkov et al., 2008) were adopted to collect self-report data on individuals’ decision-making styles.

GDMS is a validated tool for profiling individuals according to five different decision-making styles (rational, intuitive, dependent, avoidant, and spontaneous) and is composed of 25 items, for each of which the participant is asked to indicate his/her level of agreement on a 5-step Likert scale. An individual with a rational decision-making style tends to make decisions based on careful consideration and evaluation of different alternatives, because of a comprehensive and exhaustive search for information, while a person with an intuitive decision-making style is driven to make decisions based on intuitions derived from the attention paid to global aspects. The dependent decision-making style, on the other hand, is characterized by constantly seeking suggestions and advice from other people before deciding, while the avoidant style is defined by a tendency to avoid making decisions. Finally, an individual with a spontaneous decision-making style prefers to decide as quickly as possible.

The MS is a validated questionnaire consisting of 13 items (Nenkov et al., 2008) that require individuals to express their degree of agreement on a 7-step Likert scale that allows one to measure decision makers’ tendencies (i) to hold high standards for themselves and things in general (the high standard subscale), (ii) to seek better options (the alternative search subscale), and (iii) to encounter difficulties in making a choice (the decision difficulty subscale).

### 2.4 Data analysis

First, an exploratory repeated-measures ANOVA was applied to the whole sample with *Task* (2: RRT, RAT) as a within-subject independent factor and behavioral scores as dependent measures, to obtain a preliminary view of general trends within the total sample. In addition, to specifically test group differences, a further mixed ANOVA including *Group* (2: junior, senior) as a between-subject independent factor and *Task* (2: RRT, RAT) as a within-subject independent factor was applied to the behavioral scores as dependent measures.

Simple effects for significant interactions were further checked via pairwise comparisons, and Bonferroni correction was used to reduce potential biases of multiple comparisons. Furthermore, the normality of the data distribution was preliminarily assessed by checking kurtosis and asymmetry indices. The size of statistically significant effects has been estimated by computing eta squared (η^2^) indices. The threshold for statistical significance was set at *α* = 0.05.

The relationship between RRTi, and RATi and the decision-making styles has then been further explored via linear regressions: First by analyzing the whole sample in order to get a preliminary general glimpse of such relationship and, second, via subgroup analysis. Specifically, the GDMS subscale scores (rational, intuitive, dependent, avoidant, and spontaneous) and the MS subscale scores (choose between alternatives, research of options, and high standards) have been used as predictors in separate multiple linear regression stepwise models including RRTi and RATi as predicted dependent measures. Scatterplots were drawn to check for the linearity of the relationship between the predictor and dependent measures included in the regression models. Assumptions concerning the homoscedasticity, linearity, and normality of residuals were also checked by examining the scatterplot of standardized predicted values versus standardized residuals as well as the P–P plot of standardized residuals. The Durbin–Watson statistic was computed to determine the autocorrelation of the residuals, and tolerance and variance inflation indices were calculated to check multicollinearity. The effect size of the dependence relationship between the predictor and dependent variables was estimated with the R-square. Following Cohen’s (1988) rules, effect sizes are considered small when ≥0.02, medium when ≥0.13, and large when ≥0.26. The threshold for statistical significance was set at *α* = 0.05.

## 3 Results

### 3.1 Total sample

**Figure 2:**
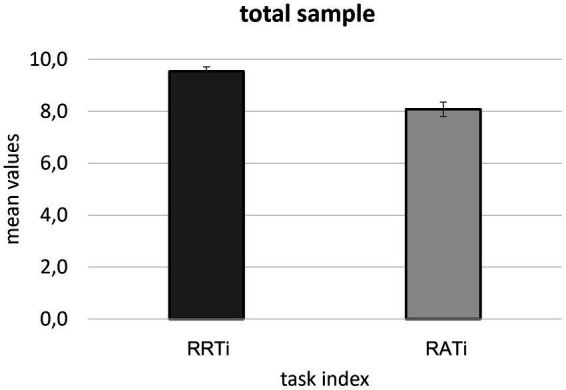
Bar graph shows the significant differences between RRTi and RATi observed in the total sample. Bars indicate the ±1 Standard Error (SE). The star (*) marks the significant difference.

From the first ANOVA performed on the total sample, a significant main effect for the *Task* factor was found [*F*(1, 60) = 21,472 *p* ≤ 0.001, *η^2^* = 0.264], for which higher behavioral scores were detected for RRTi compared to RATi (Figure 2).

The multiple linear regression model focusing on the relationship between GDMS subscale scores as predictors and the RRTi score as predicted variable highlighted the significant role of GDMS dependent scores as predictors [*F*(1, 59) = 5,232, *p* = 0.026], with a slope coefficient (β) equal to −0.29. The R^2^ value was 0.083, qualifiable as a small-to-medium effect size. The Durbin–Watson value was 2.242.

No other multiple linear regression model highlighted significant effects in the total sample.

### 3.2 Subgroup comparison

The ANOVA performed by splitting the sample into the two groups (senior and junior professionals) confirmed a significant main effect for the *Task* factor [*F*(1, 59) = 21,524, *p* ≤ 0.001, *η^2^* = 0.267], for which higher behavioral scores were detected for RRTi compared to RATi. No significant effects nor significant interaction effects were found for the Group variable.

**Figure 3:**
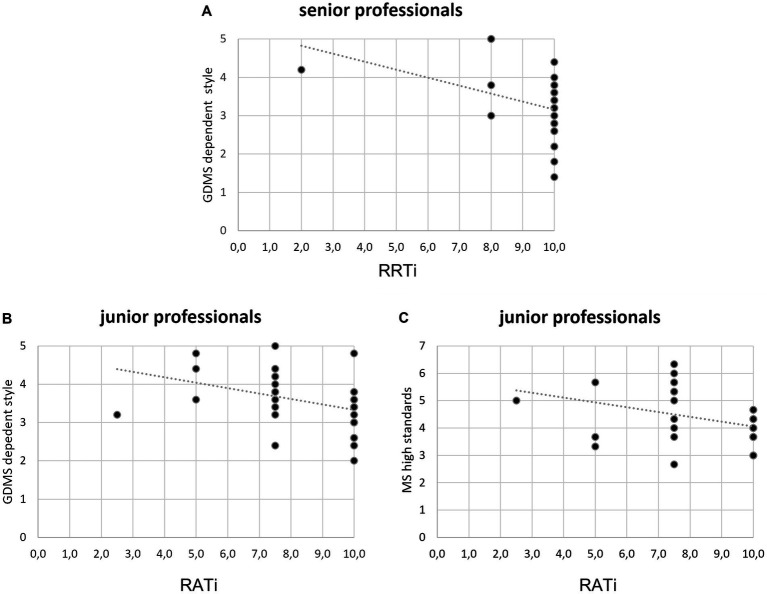
**(A–C)** Scatterplots and regression line estimates for statistically significant regression models including **(A)** GDMS-dependent style as the predictor variable and RRTi as the dependent variable in senior professional, **(B)** GDMS-dependent style as the predictor variable and RATi as the dependent variable in junior professional, **(C)** MS high standards as the predictor variable and RATi as the dependent variable in a junior professional. Straight lines represent global linear trends.

For the group of senior professionals, the multiple linear regression model focusing on the relationship between the GDMS subscale scores as predictors and the RRTi score as predicted variable showed a significant role of GDMS dependent scores as predictor [*F*(1, 27) = 4,840, *p* = 0.037], with a slope coefficient (β) equal to −0.39. The R^2^ value was 0.15, qualifiable as a medium effect size (Figure 3A). The Durbin–Watson value was 2.331.

No other multiple linear regression model highlighted significant effects in the senior professionals’ subgroup.

For the group of junior professionals, the multiple linear regression model focusing on the relationship between the GDMS subscale scores as predictors and the RATi score as predicted variable showed the significant role between predictor and dependent variable [*F*(1, 31) = 4,954, *p* = 0.034], with a slope coefficient (β) for GDMS dependent subscale equal to −0.38. The R^2^ value was 0.14, qualifiable as a medium effect size (Figure 3B). The Durbin–Watson value was 1.815.

Additionally, the multiple linear regression model focusing on the relationship between the MS subscale scores as predictors and RATi as predicted variable showed the significant role of MS high standards scores [*F*(1, 31) = 4,487, *p* = 0.043], with a slope coefficient (β) equal to −0.36. The R^2^ value was 0.13, qualifiable as a medium effect size (Figure 3C). The Durbin–Watson value was 1.813.

No other multiple linear regression model highlighted significant effects in the junior professionals’ subgroup.

## 4 Discussion

This study explored the ability of professionals to resist reframing and decoy alternatives in decision-making conditions, focusing specifically on the differences between professionals already entered the world of work and junior professionals. Two novel behavioral tasks were proposed to participants for exploring their resistance to reframing—the Resistance to Reframe Task (RRT)—and decoy alternatives—the Resistance to the Alternatives Task (RAT)—in organizational settings. Furthermore, the relationship between individual differences in decision-making styles (measured through the GDMS and MS scales) and resistance to reframing and alternatives was investigated.

The results derived from two distinct analyses will be discussed below, i.e., from a first analysis carried out on the overall sample and then from a more in-depth analysis applied to the two subgroups of professionals. The latter was carried out to highlight potential differences attributable to job seniority.

First, the whole professionals showed to be able to resist more to the reframing (RRT) than the decoy alternatives (RAT) task, without any difference between groups. Thus, on one hand, professionals demonstrated to be able to run counter a reframed condition and display a “rational,” description-invariant behavior (De Martino et al., 2006); on the other hand, they all showed a lower ability to resist multiple alternatives presented in such a way as to evoke the decoy effect. Moreover, differently from what was hypothesized no differences related to seniority were found. This result is partially in line with Slaughter et al. (2011) previously demonstrated that the ability to exploit the decoy effect did not depend on seniority, but they supposed it depended rather on the type of expertise. This evidence adds to this line of research that seniority did not impact resistance to reframing and the decoy effect. However, some differences in terms of seniority emerge if professionals’ decision-making style is taken into consideration.

In fact, by including in this framework the individual style that each person adopts in making a decision, it was observed how higher scores at the GDMS-dependent subscale predict lower RRT scores. Thanks to the second-level subgroup analysis, it emerged that such an effect was attributable mainly to the group of senior professionals and not to junior ones. This means, that with advancing age, having a predominance of dependent decision-making style, that is characterized by constantly seeking suggestions and advice from other people before deciding (Scott and Bruce, 1995; Thunholm, 2004; Gambetti et al., 2008), might reduce the resistance to the reframe and can lead to making decisions more dependent on the context (and therefore on the frame) or dependent on comparisons with other people (typical of this decision-making style).

Interestingly, some peculiarities related to decision-making style and resistance to alternatives were found also in the group of junior professionals. Indeed, two main decision-making profiles were demonstrated to predict lower RAT scores in the group of junior professionals: One connoted by high MS high standards scores, and one connoted by high GDMS dependent scores. This result demonstrated that, even in the group of junior professionals, it is always a context-dependent decisional profile (comparing with others to receive advice on how to decide or considering others as a comparison standard to be overcome) that makes the resistance to multiple decoy alternatives more complex.

The reason why in junior professionals, a context-dependent decision-making profile predicts lower RAT scores, and in seniors, the same profile predicts lower RRT scores (together with a generally lower ability to resist RAT, rather than RRT, regardless of the decision-making style, as demonstrated by the ANOVA) must be investigated also taking into consideration the role of EFs, examining whether a reduction in cognitive control toward these biases also occurs in this specific case.

Another recent study (Tommasi et al., 2023) explored the presence of bias in professionals and demonstrated that entrepreneurs exhibit higher levels of under/overconfidence (i.e., self-confidence in taking decisions) than managers and specifically showed a marked presence of this bias among entrepreneurs at younger ages. Therefore, both higher levels of expertise and seniority in terms of age require thorough investigation in the context of resistance to decision biases.

To conclude, this study suggests that professionals know how to “keep the tiller straight” in organizations, especially when facing reframing conditions, rather than decoy alternatives; however, the predominance of dependent decision-making styles (both for senior and junior professionals), and the tendency to hold high standards in decisions (mainly for juniors) could undermine their resistance capacity and make them vulnerable to these covert influence tactics.

Although our current study is one of the first studies investigating the construct of resistance to decision bias in professional contexts, and their relationship to decision-making styles, it is not without caveats. Among all limitations, the presence of only behavioral data would benefit from the integration of neurophysiological data to explore professionals’ EFs and increase the validity and generalizability of current results.

## Data availability statement

The raw data supporting the conclusions of this article will be made available by the authors, without undue reservation.

## Ethics statement

The studies involving humans were approved by Department of Psychology of the Catholic University of the Sacred Heart, Milan, Italy. The studies were conducted in accordance with the local legislation and institutional requirements. The participants provided their written informed consent to participate in this study.

## Author contributions

LA: Validation, Visualization, Writing – original draft, Writing – review & editing. CA: Data curation, Formal analysis, Investigation, Writing – review & editing. DC: Data curation, Formal analysis, Methodology, Writing – review & editing. MB: Conceptualization, Methodology, Project administration, Resources, Supervision, Validation, Writing – review & editing.
